# Rate of acyl migration in lysophosphatidylcholine (LPC) is dependent upon the nature of the acyl group. Greater stability of sn-2 docosahexaenoyl LPC compared to the more saturated LPC species

**DOI:** 10.1371/journal.pone.0187826

**Published:** 2017-11-08

**Authors:** Dhavamani Sugasini, Papasani V. Subbaiah

**Affiliations:** 1 Section of Endocrinology, Department of Medicine, and Department of Biochemistry & Molecular Genetics, University of Illinois at Chicago, Illinois, United States of America; 2 Jesse Brown VA Medical Center, Chicago, Illinois, United States of America; Universidade de Sao Paulo Instituto de Quimica, BRAZIL

## Abstract

Several previous studies reported that sn-2 acyl lysophosphatidylcholines (LPCs) undergo rapid isomerization due to acyl migration, especially at physiological pH and temperature. However, these studies have been carried out using mostly sn-2 palmitoyl LPC, whereas the naturally occurring sn-2 LPCs are predominantly unsaturated. In this study, we investigated the acyl migration in four naturally occurring sn-2 acyl LPCs (sn-2 16:0, sn-2 18:1, sn-2 20:4, and sn-2 22:6) stored at various temperatures in aqueous or organic solvents, employing LC/MS to analyze the isomer composition. At 37°C and pH 7.4, the order of acyl migration rates (from sn-2 to sn-1) in aqueous buffer was 16:0 LPC> 18:1 LPC> 20:4 LPC> 22:6 LPC. The rate of isomerization of sn-2 16:0 LPC was 2–5 times greater than that of sn-2 22:6 under these conditions. Complexing the LPCs to serum albumin accelerated the acyl migration of all species, but sn-2 22:6 LPC was least affected by the presence of albumin. The migration rates were lower at lower temperatures (22°C, 4°C, and -20°C), but the differences between the LPC species persisted. All the sn-2 acyl LPCs were more stable in organic solvent (chloroform: methanol, 2:1 v/v), but the effect of the acyl groups on acyl migration was evident in the solvent also, at all temperatures. Storage of sn-2 22:6 LPC at -20°C for 4 weeks in the organic solvent resulted in about 10% isomerization, compared to 55% isomerization for sn-2 16:0. These results show that the sn-2 polyunsaturated LPCs can be stored at -20°C or below for several days without appreciable isomerization. Furthermore, they demonstrate that the sn-2 polyunsaturated LPCs generated *in vivo* are much more stable under physiological conditions than previously assumed.

## Introduction

Phosphoglycerides in the mammalian tissues have predominantly a saturated fatty acid at the sn-1 position and an unsaturated fatty acid at the sn-2 position. The two acyl groups turnover independently of each other because of the presence of position-specific phospholipases and acyltransferases that remodel the phospholipids [1]. Therefore, both sn-1 acyl and sn-2 acyl lysophosphatidylcholines (LPCs) are formed from the phosphatidylcholines (PCs) *in vivo*. However, several studies have reported that there is a rapid isomerization of sn-2 acyl isomer to the more stable sn-1 acyl isomer [2–5]. The ratio of sn-1 acyl isomer to sn-2 acyl isomer has been shown to be about 9:1 at equilibrium in aqueous solutions [2], The acyl migration is greatly accelerated at or above physiological pH, and at higher temperatures [3]. Chromatography on silicic acid or alumina columns is also known to increase the acyl migration [6–8], but the isomerization can be minimized by storing the compounds in the acidic pH [2,3] and at low temperatures [3]. It should, however, be pointed out that most of these studies were carried out with sn-2 palmitoyl (16:0) LPC, whereas the naturally occurring sn-2 acyl LPCs are predominantly polyunsaturated [9]. Furthermore, despite the reported rapid isomerization of the sn-2 acyl LPC s *in vitro*, there is no evidence for the presence of significant percentage of polyunsaturated acyl groups at the sn-1 position of natural phosphoglycerides except in the brain [10], as would be expected if the isomerized LPCs are acylated by the ubiquitous acyltransferases [1]. It is therefore important to determine whether the naturally formed polyunsaturated LPCs also exhibit acyl migration to the same extent as sn-2 16:0 LPC. Previous studies suggested that the metabolic effects of sn-2 docosahexaenoyl (DHA, 22:6) LPC are different from those of the sn-1 isomer [11,12]. However, our recent studies showed that both isomers of LPC-DHA are absorbed and incorporated into the brain to the same extent [13]. It is therefore important to determine whether the lack of difference between the two isomers is because of the rapid isomerization of the sn-2 acyl isomer to sn-1 acyl isomer. In this study, we have compared the rate of isomerization of sn-2 22:6 LPC with that of more saturated LPC species under various conditions *in vitro*. The results presented here clearly show that in contrast to the previously reported rapid isomerization of sn-2 16:0 LPC, the isomerization of sn-2 22:6 LPC is much slower under physiological conditions. The acyl migration in sn-2- 18:1 and sn-2-20:4 LPCs fell between the two extremes. These results further indicate that the relative stability of the polyunsaturated LPCs enables the preservation of polyunsaturated fatty acids (PUFA) at the sn-2 position *in vivo* during the metabolic turnover of polyunsaturated PC species.

## Materials and methods

### Materials

All synthetic PCs used to produce the sn-1 and sn-2 acyl LPCs (16:0–16:0, 16:0–18:1, 16:0–20:4, and 16:0–22:6 PCs) were purchased from Avanti Polar Lipids (Alabaster, AL). Immobilized *Mucor meihei* lipase (Lipozyme, 30 milliunits/ mg), methanol, acetonitrile, ultra-pure water, ammonium formate, formic acid, chloroform, all of MS grade, were purchased from Sigma Aldrich (St Louis, MO).

### Preparation of LPC isomers

The sn-2 acyl LPCs were prepared by the hydrolysis of the corresponding PCs with *Mucor meihei* lipase, which is specific for the sn-1 ester linkage [14]. Briefly, 100 mg of PC (16:0–16:0, 16:0–18:1, 16:0–20:4 or 16:0–22:6) was dissolved in 2 ml of ethanol: water (95:5, v/v) in a 20 ml glass scintillation vial, and 200 mg (6 units) of Lipozyme (immobilized M*ucor meihei* lipase) was added to the solution and vortexed thoroughly for 1 min. To minimize the oxidation of DHA, the sample was flushed with nitrogen, and the reaction was carried out in the dark at 37°C for 24 h, with continuous shaking in a metabolic shaker. The reaction mixture was then dried under nitrogen, and extracted with 8 ml of ethanol: water: hexane (2:1:1, v/v). After centrifugation at 1500 rpm for 10 min, the upper hexane layer, containing the released free fatty acids, was removed by aspiration. The lower layer, containing the enzyme, LPC and any unhydrolyzed PC was extracted by Bligh and Dyer procedure [15]. An aliquot of the lipid extract was spotted on the TLC plate and PC and LPC separated using chloroform: methanol: water (65:25:4 v/v) as the developing solvent. The spots corresponding to PC and LPC were detected by exposure to iodine vapors and the lipid phosphorus was determined in the spots [16]. Typically, we found at least 95% of the lipid phosphorus in LPC after 24 h reaction, and therefore the samples were used without further purification. The samples were routinely stored in chloroform: methanol (2:1 v/v at -20°C).

The sn-1 acyl isomers of the various LPCs were prepared by the exposure of the methanolic solutions of the corresponding sn-2 acyl LPCs to ammonia vapors at room temperature for 48 h in the dark, under nitrogen [14]. More than 90% of the sn-2 acyl LPC was converted to the sn-1 acyl isomer under these conditions. These compounds were used to determine the relative retention times of the sn-1 acyl and sn-2 acyl isomers, as well as their ionization efficiency in LC/MS/MS (liquid chromatography/tandem mass spectrometry).

### Study of acyl migration during storage

The stability of the sn-2 acyl LPCs was determined during storage in both aqueous and organic solvents. For the preparation of the aqueous solution, the solvent was evaporated under nitrogen, and 5 ml of 10 mM Tris-HCl buffer, pH 7.4 was added to the dried lipid (10 mg) and sonicated 3 times at 30% power (for 1 min each) at 4°C in a VCX Vibracell sonicator. All LPCs yielded a clear solution at this concentration. Aliquots of the solution were then stored at -20°C, 4°C, 22°C, and 37°C for various periods of time as indicated, before subjecting them to LC/MS analysis. All the samples were first brought to room temperature (22°C), diluted with equal volume of methanol and injected into the LC/MS system. For storage in the organic solvent, the samples were dissolved at a concentration of 1 mg/ml in chloroform: methanol (2:1, v/v) and stored at the same temperatures as above, before LC/MS/MS analysis. This solvent was chosen because it is routinely used for storage of lipids in our laboratory.

### Effect of serum albumin on acyl migration

For testing the effect of albumin on acyl migration, freshly prepared sn-2 acyl LPC (10 mg) was first dissolved in 50 μl ethanol, and diluted to 5 ml with 0.1% bovine serum albumin in phosphate buffered saline (pH 7.4). The sample was sonicated with a probe sonicator (Vibracell, at 30% power) for 60 sec before incubation at 37°C. At the indicated time point, a 10 μl aliquot was taken out and deproteinated by adding 190 μl of acidified methanol (pH 4.0, adjusted with 10% TCA). The solution was sonicated for 1 min in a bath sonicator and the precipitated protein was removed by centrifugation. An aliquot of the supernatant was then analyzed by LC/MS/MS as described below.

### Analysis of LPC regioisomers by LC/ MS/MS

sn-1-acyl and sn-2-acyl LPC isomers were analyzed by LC/MS/MS using an AB Sciex QTRAP 6500 mass spectrometer coupled with Agilent 2600 UPLC system, essentially according to Koistinen et al [17]. The aqueous LPC samples (2 mg/ ml) were first diluted with equal volume of methanol, and 10 μl of the sample (containing about 10 μg test LPC) was injected onto the UPLC column. Chromatographic separation was carried out using an Atlantis^®^ HILIC column (2.1 mm × 150 mm, 3 μm) (Waters corporation, Milford, MA). Mobile phase A consisted of 50 mM ammonium formate in water containing 0.2% formic acid (v/v) whereas mobile phase B was acetonitrile with 0.2% formic acid. The chromatographic conditions were as follows: initial concentration of mobile phase A was 5%, which was increased to 15% in 1 min, followed by a linear increase to 30% in 10 min, and to 50% in another 5 min, and finally the equilibration of the column with 5% A for an additional 10 min (total run time, 26 min). The flow rate was 500 μl/ min and the column temperature was maintained at 40°C. Electrospray-mass spectrometry was performed in positive multiple reaction monitoring (MRM) mode for the quantitative analysis of LPC isomers (see Table 1 for the MRM values). The source temperature was set at 300°C, the ion spray voltage at 5.5 KV, and the collision energy at 30 eV. Mass spectra were acquired and recorded with Analyst software (AB Sciex). The MRM data was processed using the quantitation wizard in Analyst software (AB Sciex, Concord, Canada). Equimolar mixture of freshly prepared sn-2 acyl and sn-1 acyl isomers was analyzed under the above conditions to determine the relative ionization efficiencies. Since in all cases the area counts of the two isomers were within 5% of each other (results not shown), the area counts of the peaks were used to calculate the percentage composition of the two LPC isomers. Identification of the regioisomers was confirmed by the product ion spectra of the peaks [17] with the collision energy set at 27 eV.

**Table 1:** MRM transitions used for LC-MS analysis of lysophospholipids.

Compound	Q1(m/z)	Q3 (m/z)
LPC (16:0)	496.3	184.1
LPC (18:1)	522.4	184.1
LPC (20:4)	544.3	184.1
LPC (22:6)	568.3	184.1

## Results

### Acyl migration in aqueous solution at 37°C, pH 7.4

**Fig 1:**
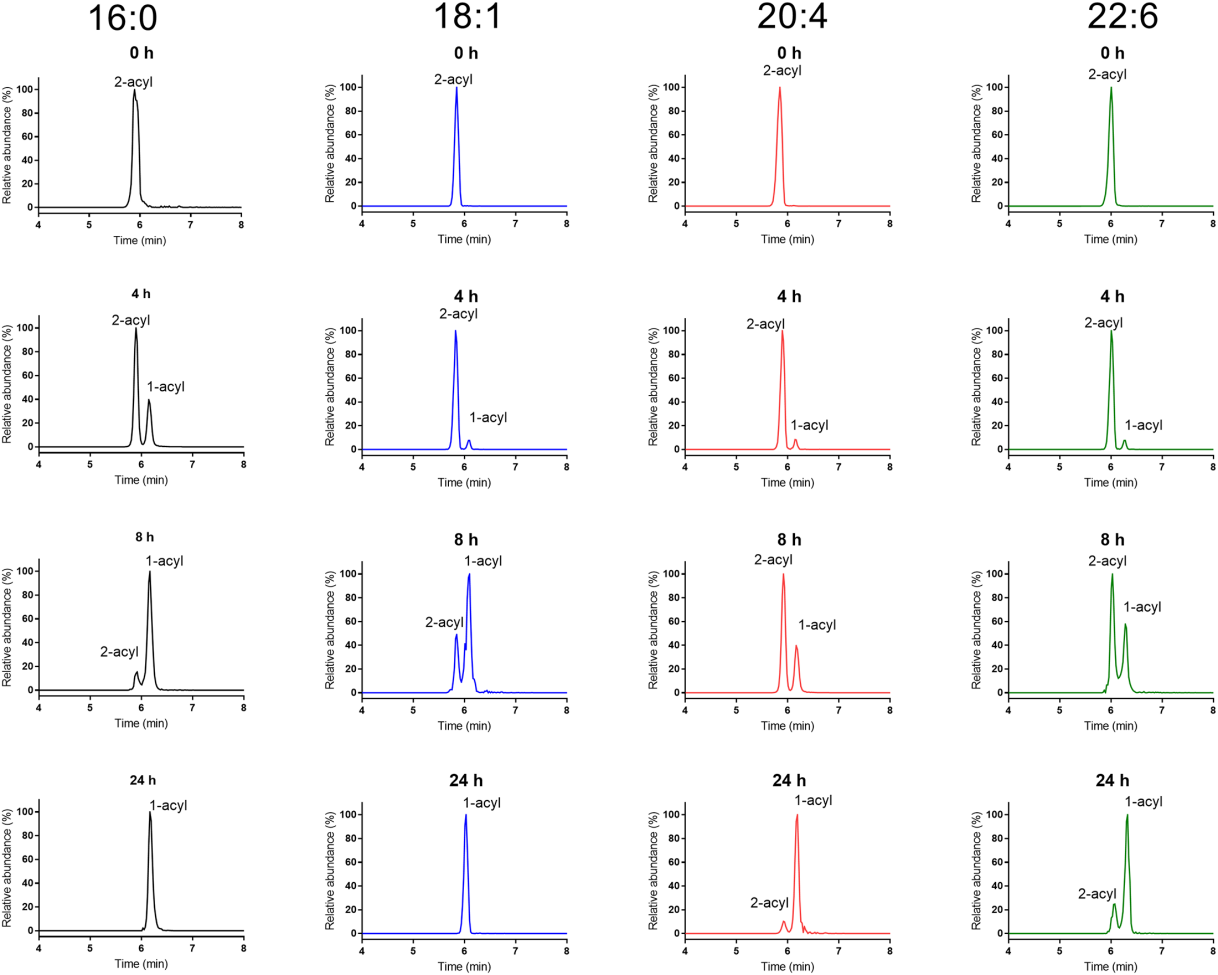
Selected LC/MS chromatograms of sn-2 acyl LPC species incubated at 37°C in Tris-HCl buffer, pH 7.4. The aqueous solution of each sn-2 acyl LPC (2 mg/ml) was incubated at 37°C, and aliquots were taken out at the indicated time. The samples were diluted with equal volume of methanol, and analyzed by LC/MS/MS in the MRM mode, as described in Methods.

**Fig 2:**
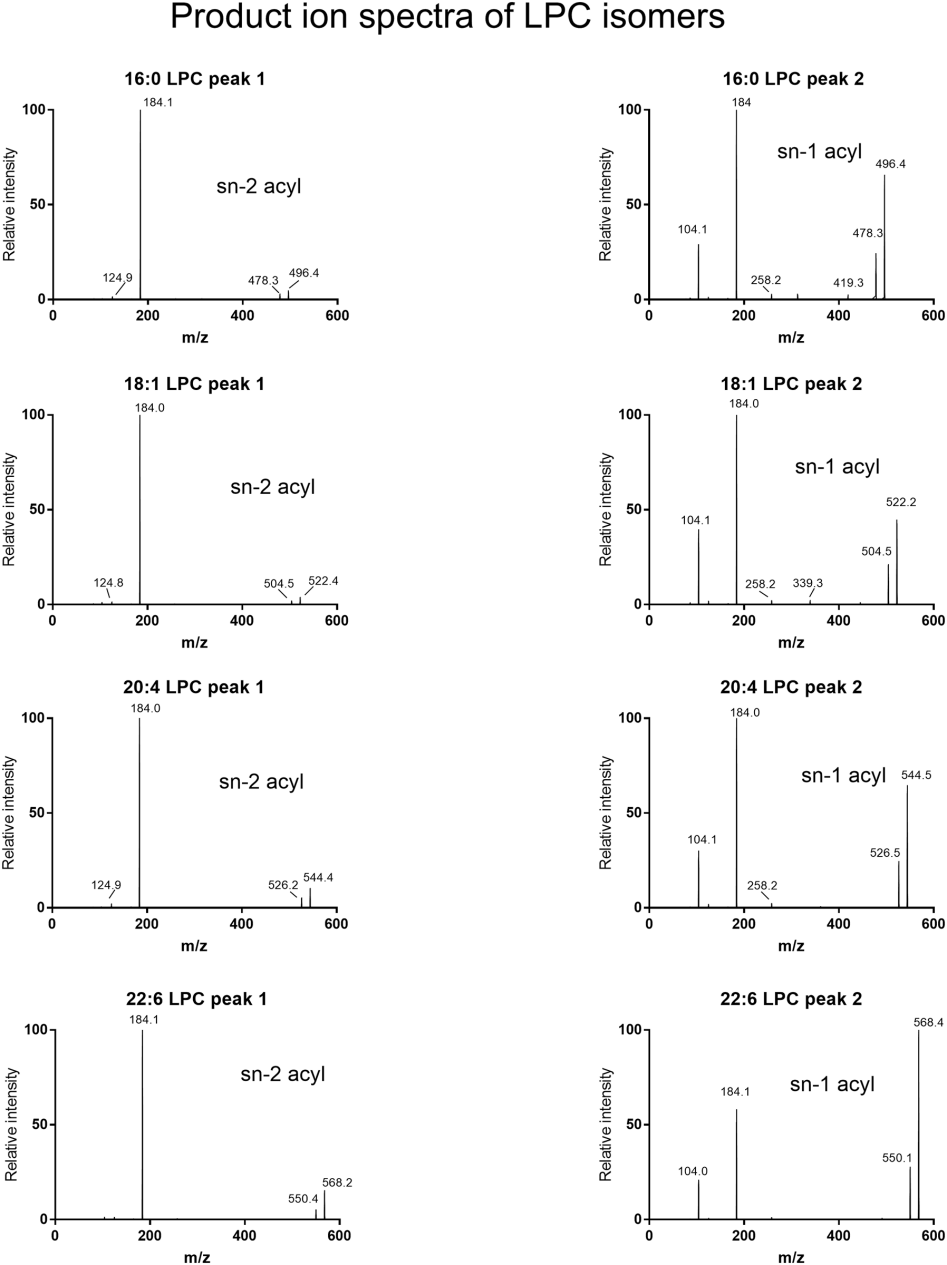
Identification of the regioisomers of LPC from the product ion spectra. The product ion spectra were obtained for the early eluting (peak 1) and late eluting (peak 2) isomers of each LPC as described in Methods. The intensity of the molecular ion as percentage of that of phosphocholine ion (m/z 184) differentiates the two regioisomers, with the sn-1 acyl isomer showing much higher percent of molecular ion [17]. The relative intensities of the molecular ion, expressed as percent of phosphoryl choline ion (m/z 184.1) for the various LPCs are as follows: 16:0 LPC (4.7% for peak 1 and 65.5% for peak 2); 18:1 LPC (3.7% for peak 1 and 44.8% for peak 2); .20:4 LPC (10.2% for peak 1 and 60.5% for peak 2); 22:6 LPC (15.3% for peak 1, and,172% for peak 2). In addition, the choline fragment ion (m/z 104.1) is completely absent from the sn-2 acyl isomers, but was prominently present in all the sn-1 acyl isomers.

To determine the rate of acyl migration at physiological temperature and pH, we prepared aqueous solutions of sn-2 acyl LPCs in Tris-HCl buffer, pH 7.4, incubated them at 37°C for various periods and fractionated them by LC/MS immediately, as described in Methods. The LPCs in the aqueous solution were mixed with equal volume of methanol and injected directly (10 μl) onto the UPLC column. Selected chromatograms of sn-2 16:0, sn-2 18:1, sn-2 20:4, and sn-22:6 LPCs from one representative experiment are shown in Fig 1. In all cases, the sn-2 acyl isomer eluted earlier than the sn-1 acyl isomer, which is confirmed by the product ion spectrum of each peak (Fig 2). The early eluting peak in all cases showed much lower ratio of parent ion to phosphoryl choline ion (m/z 184.1) compared to the later eluting peak, confirming that the first peak is the sn-2 acyl isomer [17]. Furthermore, the choline fragment ion (m/z 104.1) was prominent in sn-1 acyl isomer, but was absent in the sn-2 acyl isomer, as predicted from the fragmentation pathways [18]. In accordance with previous studies [2,3], sn-2 16:0 LPC isomerized to the sn-1 acyl isomer rapidly at 37°C, with only about 13% of sn-2 isomer remaining after 8 h at 37°C. The acyl migration, however, was much slower for the polyunsaturated LPCs (20:4 and 22:6), with > 60% of the sn-2 acyl isomer remaining after 8 h. The acyl migration in sn-2 18:1 LPC was intermediate between the two extremes, with about 29% of the sn-2 acyl isomer remaining after 8 h. After 24 h at 37°C, no detectable sn-2 16:0 LPC or sn- 2 18:1 was present, whereas 10% of sn-2 20:4 and 22% of sn-2 22:6 LPCs remained unchanged. These results show that the rate of acyl migration at physiological pH and temperature is highly dependent upon the structure of the acyl group at the sn-2 position of LPC.

### Effect of temperature on acyl migration in aqueous buffer, pH 7.4

**Fig 3:**
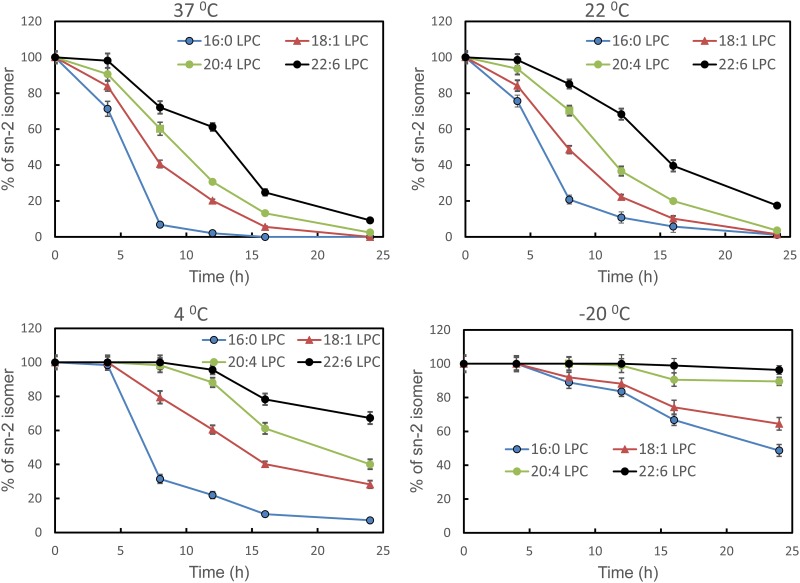
Stability of aqueous dispersions of sn-2 acyl LPCs at various temperatures. The sn-2 acyl LPCs were dispersed in Tris-HCl buffer, pH 7.4 at a concentration of 2 mg/ml and incubated under nitrogen in the dark at the indicated temperature for 24 h. Aliquots were taken out at 0 h, 4 h, 8 h, 12 h, 16 h, and 24 h, brought to room temperature, diluted with equal volume of methanol and subjected to LC/MS/MS analysis as described in Methods. The percentage of the sn-2 acyl isomer remaining was calculated from the area counts of the two isomers. The values shown are mean ± SD of 3 separate experiments.

The isomerization of all the sn-2 LPC species was then determined following their incubation at varying temperatures (-20°C, 4°C, 22°C, and 37°C) in 10 mM Tris-HCl buffer, pH 7.4, in dark. All samples were brought to room temperature (22°C), diluted with an equal volume of methanol, and then analyzed by LC/MS/MS. The percentages of sn-2 isomer remaining at each time point are shown in Fig 3 (mean ± SD of 3 separate analyses). As expected, the acyl migration was much slower at the lower temperatures, but the differences between the LPC species were still apparent. After 24 h, the sn-2 16:0 LPC underwent isomerization significantly at all temperatures, except at -20°C. At 4°C, the polyunsaturated LPCs (20:4 and 22:6) were almost completely stable for 8 h, whereas about 20% of sn-2 18:1 and 69% of sn-2 16:0 LPC were converted to the sn-1 isomers. At room temperature (22°C), about 80% of sn-2 16:0 isomerized in 8 h, whereas only about 15% of sn-2-22:6 LPC isomerized. After storage for 24 h at -20°C, about 96% of sn-2-22:6 LPC remained unchanged, whereas over 50% of sn-2-16:0 LPC was converted to the sn-1 isomer.

### Acyl migration in organic solvent

**Fig 4:**
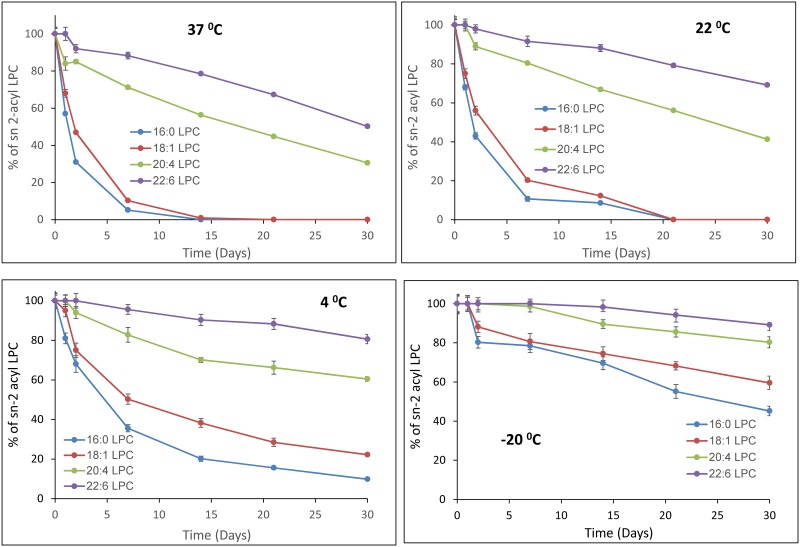
Stability of sn-2 acyl LPCs in organic solvent. The sn-2 acyl LPCs were dissolved in chloroform: methanol (2:1 v/v) at a concentration of 1 mg/ml, and stored at the indicated temperature for various periods. Aliquots were taken out at the indicated time points, brought to room temperature (22°C), and subjected to LC/MS, as described in Methods. The percentage of the sn-2 acyl isomer remaining was calculated from the area counts of the two isomers. The values shown are mean ± SD of 3 separate experiments.

The acyl migration in the sn-2 acyl LPC isomers was also determined in an organic solvent, to assess the stability of these compounds during long-term storage in the solvents. As shown in Fig 4 (mean ± SD of 3 experiments), the acyl migration was much slower in chloroform: methanol (2:1 v/v) compared to the aqueous dispersion, at all temperatures. When stored in the solvent at -20°C, only about 10% of sn-2-22:6 LPC was isomerized in 4 weeks, whereas about 20% of sn-2-20:4 LPC was isomerized. However, about 40% of sn-2-18:1 LPC and 55% of sn-2-16:0 LPC were converted to their sn-1 acyl isomers even at -20°C in 4 weeks. In the refrigerator (4°C) about 20% of sn-2-22:6, 40% of sn-2-20:4, 78% of sn-2-18:1, and 90% of sn-2-16:0 isomerized to the respective sn-1 isomers in 4 weeks. Even at room temperature (22°C), the sn-2-20:4 and sn-2-22:6 LPCs were completely stable for one day if stored in chloroform: methanol (2:1, v/v) in the dark. However, about 25% of sn-2-18:1 LPC and 32% of sn-2-16:0 isomerized under the same conditions.

### Effect of serum albumin on acyl migration

**Fig 5:**
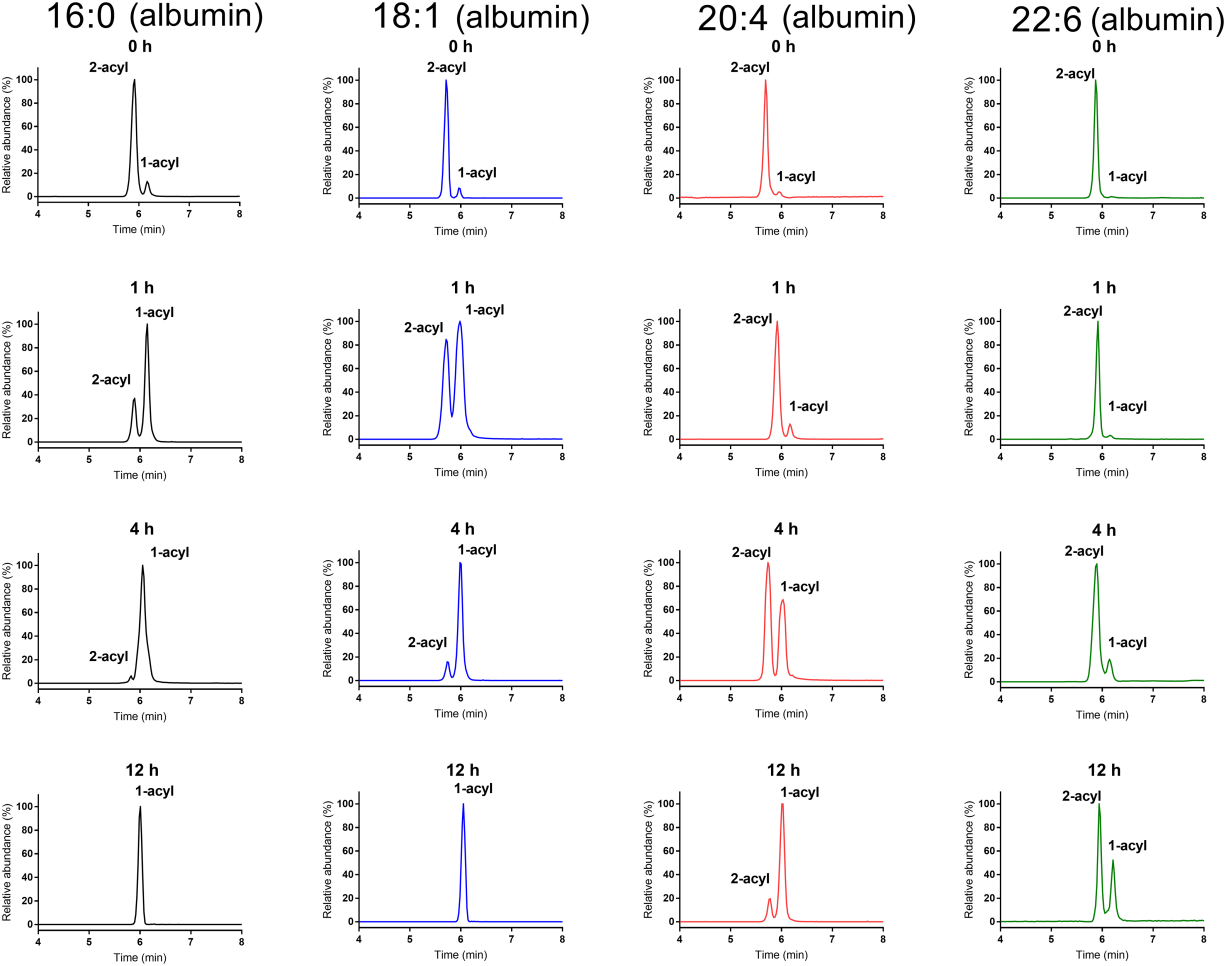
Effect of serum albumin on the isomerization of sn-2 acyl LPC at 37°C. Freshly prepared sn-2 acyl LPCs were complexed with BSA in PBS, pH 7.4, and incubated at 37°C for the indicated periods. The isomer composition was then determined by LC/MS/MS as described in Methods. Chromatograms of only selected time periods are shown. The time course of the isomerization of each LPC is shown in Fig 6.

Since most of the LPC in plasma is associated with serum albumin, it is important to determine whether the differences in acyl migration persist when the LPCs are bound to albumin. Previous studies by Okudaira et al [9] showed that the acyl migration in sn-2 18:1 lysophosphatidylserine is accelerated greatly by the presence of albumin. Therefore, we repeated the above studies with the four species of sn-2 acyl LPC in aqueous buffer in the presence of 0.1% bovine serum albumin. The albumin-bound LPCs (pH 7.4, in PBS) were incubated at 37°C for various periods, extracted with acidified methanol, and analyzed by LC/MS/MS as described above. As shown in Fig 5, the acyl migration in sn-2 16:0 LPC was dramatically accelerated in the presence of albumin, compared to the rate in pure buffer (Fig 1). Thus only 3% of the sn-2 16:0 isomer remained after 4 h at 37°C, compared to about 70% in the absence of albumin. The acyl migration in sn-2 18:1 LPC was also markedly increased by the presence of albumin, compared to the rate in the absence of albumin. Only 12% of the sn-2 18:1 isomer remained unchanged after 4 h in the presence of albumin, compared to 94% in the absence of albumin (Fig 1). The polyunsaturated LPCs were more stable than the more saturated species even in presence of albumin. Thus about 54% of sn-2 20:4 LPC, and 88% of sn-2 22:6 LPC were unchanged after 4 h at 37°C. Remarkably, over 60% of sn-2 22:6 LPC isomer remained unchanged even after 12 h 37°C, and 15% remained after 24 h (not shown). These results further confirm the greater stability of polyunsaturated LPCs, especially the sn-2 22:6 LPC under physiological conditions.

**Fig 6:**
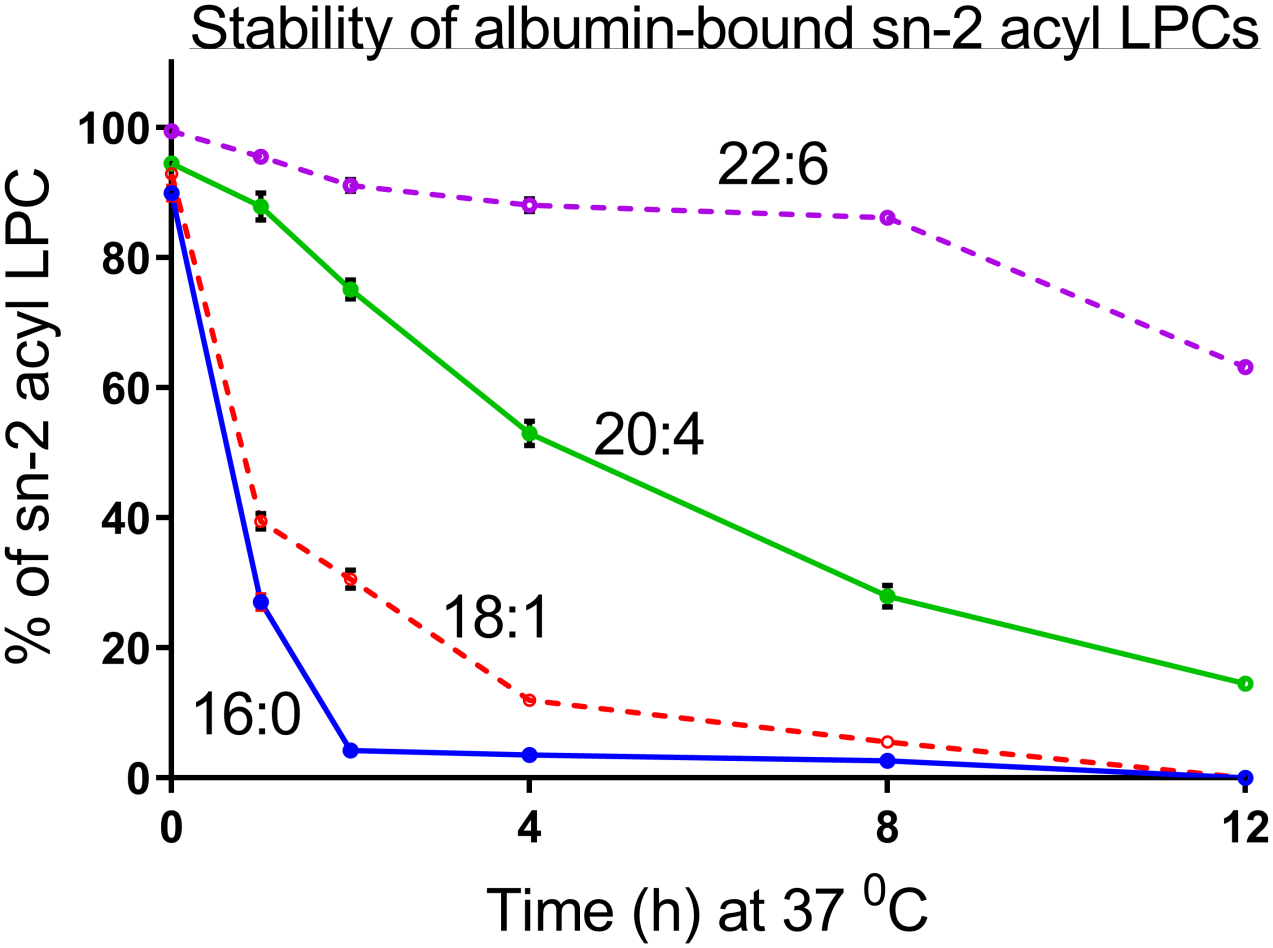
Time course of isomerization of albumin-bound sn-2 acyl LPCs at 37°C. Freshly prepared sn-2 acyl LPCs were complexed with 0.1% BSA in PBS buffer at pH 7.4, and incubated at 37°C for the indicated periods of time. The percent of the sn-2 acyl isomer remaining after each time point was determined by LC/MS/MS. The values shown are mean ± SD of 3 separate analyses. Note that the error bars too small to be visible at some points.

## Discussion

The sn-2 acyl LPCs generated *in vivo* are predominantly polyunsaturated because of the well-known preferential distribution of PUFA in the sn-2 position of the natural PCs. We have previously reported that there are at least three different enzymes, which could generate sn-2 polyunsaturated LPCs in the plasma. These are endothelial lipase [19], hepatic lipase [20], and lecithin-cholesterol acyltransferase [21]. In addition, a calcium-independent phospholipase A (iPLA_2_γ) is known to generate polyunsaturated LPCs in the tissues [22]. The presence of significant amounts of polyunsaturated LPCs in the plasma has been reported by several studies [5,9,23,24]. However, most investigations on the acyl migration of sn-2 acyl LPC employed a saturated (sn-2 palmitoyl) LPC [2–4], and they all showed a rapid migration of the acyl group from sn-2 to sn-1 position, especially at physiological pH and temperature [2,3]. A very rapid isomerization of labeled sn-2 18:2 LPC in plasma was also reported by Croset et al [5]. The results presented here, however, show that the phenomenon of rapid acyl migration is not applicable to the polyunsaturated LPCs, since they isomerize much more slowly, compared to sn-2 palmitoyl or oleoyl LPC, under physiological conditions.

The acyl migration in glycerolipids most likely involves the formation of a five member cyclic intermediate which is formed by the nucleophilic attack on the carbonyl carbon at sn-2 by the free hydroxyl group of sn-1 position [2]. The subsequent opening of the ring leads to the predominant formation of the thermodynamically more stable sn-1 acyl isomer, compared to the sn-2 acyl isomer. It is possible that the presence of a long chain PUFA at the sn-2 position results in steric hindrance for the formation of cyclic intermediate, and therefore the acyl migration is much slower. Although the effect of chain length on the acyl migration was previously studied in monoacylglycerols [25], the acyl groups investigated in that study included only saturated fatty acids up to 16 carbon chain length. The present study is the first to investigate the effect of naturally occurring acyl groups on the rate of acyl migration in lipids. It may be pointed out that despite the reported rapid acyl migration of the sn-2 acyl group in LPC, the polyunsaturated LPCs isolated from the tissues have been shown to be predominantly of sn-2 acyl configuration [9], supporting the relative stability of these compounds under physiological conditions. Previous studies also reported that the acyl migration in lysophospholipids is affected significantly by the nature of the head group, the rate of migration being significantly higher in lysophosphatidyl ethanolamine, compared to the other lysophospholipids [9]. However, these studies were carried out with sn-2 18:1 lysophospholipids, which may not represent the rates of acyl migration in the naturally occurring sn-2 polyunsaturated lysophospholipids. In fact, the 18:1 was distributed equally between the sn-1 and sn-2 positions of natural lysophospholipids, whereas the distribution of 20:4 and 22:6 was predominantly at the sn-2 position [9]. Although our studies are carried out only with LPCs, these results could apply to all lysophospholipids, since the 20:4 and 22:6 in all the lysophospholipids isolated from the tissues are predominantly in the sn-2 position [9]. A comparison of our results with the effect of the head group reported by Okudaira et al [9] also suggests that the effect of the long chain polyunsaturated acyl group is greater than the effect of the head group on the rate of acyl migration.

The relative stability of sn-2 20:4 and sn-2 22:6 lysophospholipids may be physiologically significant in maintaining the positional distribution of the fatty acids *in vivo*. As mentioned above, several enzymes could generate sn-2 polyunsaturated LPCs in the plasma and tissues. A rapid isomerization of these LPCs would theoretically result in a scrambling of the fatty acid distribution in phospholipids because of the subsequent esterification of LPCs by the acyltransferases [1]. However, there is very little evidence for the presence of significant amounts of sn-1 polyunsaturated phospholipids in most tissues except brain [10] (which has significant amounts of di-22:6 PC [13]), supporting the relative stability of the sn-2 polyunsaturated lysophospholipids under physiological conditions. The studies by Lagarde and coworkers showed that significant amounts of 20:4 and 22:6 LPCs are present in normal plasma, and also that they are the preferred carriers of PUFA through the blood brain barrier [5,26,27]. These workers have also postulated that the sn-2 acyl LPCs are preferred over the corresponding sn-1 acyl isomers for transport of PUFA through the blood brain barrier, although this was not experimentally verified. They proposed the use of the sn-1 acetylated derivative of sn-2 22:6 LPC to preserve the PUFA at the sn-2 position [28]. The relative stability of the sn-2 isomers under physiological conditions, even when bound to albumin, as demonstrated here, suggests that this derivatization of LPC may not be necessary for conserving the sn-2 configuration of polyunsaturated LPCs.

In our previous studies on the absorption of dietary DHA compounds, we have compared the absorption of micellar free DHA with that of micellar LPC-DHA in lymph-duct cannulated rats [29]. Although we prepared sn-2 DHA LPC for this purpose, we assumed, based on published reports on sn-2 16:0 LPC, that it largely isomerized to sn-1 22:6 LPC during the storage and micelle preparation. The results presented here show that this assumption may not be correct. It is therefore possible that we have used predominantly sn-2 DHA LPC in the micelle, although some isomerization would have occurred during storage, micelle preparation, and during the absorption at 37°C in pH 7.4 buffer (6 h. However, our recent studies show that there is no significant difference between the two LPC-DHA isomers in their metabolic fate in the brain, or in their ability to improve memory [13].
